# Predicting per-lesion local recurrence in locally advanced non-small cell lung cancer following definitive radiation therapy using pre- and mid-treatment metabolic tumor volume

**DOI:** 10.1186/s13014-020-01546-y

**Published:** 2020-05-19

**Authors:** Michael S. Binkley, Julie L. Koenig, Mehr Kashyap, Michael Xiang, Yufei Liu, Quaovi Sodji, Peter G. Maxim, Maximilian Diehn, Billy W. Loo, Michael F. Gensheimer

**Affiliations:** 1 Department of Radiation Oncology, Stanford University School of Medicine and Stanford Cancer Institute, 875 Blake Wilbur Dr MC 5847, Stanford, CA 94305 USA; 2 Department of Radiation Oncology, Indiana University School of Medicine, Indianapolis, IN USA; 3 Institute for Stem Cell Biology & Regenerative Medicine, Stanford University School of Medicine, Stanford, California USA

**Keywords:** FDG-PET, Metabolic tumor volume, Lung cancer

## Abstract

**Background:**

We evaluated whether pre- and mid-treatment metabolic tumor volume (MTV) predicts per lesion local recurrence (LR) in patients treated with definitive radiation therapy (RT, dose≥60 Gy) for locally advanced non-small cell lung cancer (NSCLC).

**Methods:**

We retrospectively reviewed records of patients with stage III NSCLC treated from 2006 to 2018 with pre- and mid-RT PET-CT. We measured the MTV of treated lesions on the pre-RT (MTV_pre_) and mid-RT (MTV_mid_) PET-CT. LR was defined per lesion as recurrence within the planning target volume. Receiver operating characteristic (ROC) curves, cumulative incidence rates, and uni- and multivariable (MVA) competing risk regressions were used to evaluate the association between MTV and LR.

**Results:**

We identified 111 patients with 387 lesions (112 lung tumors and 275 lymph nodes). Median age was 68 years, 69.4% were male, 46.8% had adenocarcinoma, 39.6% had squamous cell carcinoma, and 95.5% received concurrent chemotherapy. Median follow-up was 38.7 months. 3-year overall survival was 42.3%. 3-year cumulative incidence of LR was 26.8% per patient and 11.9% per lesion. Both MTV_pre_ and MTV_mid_ were predictive of LR by ROC (AUC = 0.71 and 0.76, respectively) and were significantly associated with LR on MVA (*P* = 0.004 and *P* = 7.1e-5, respectively). Among lesions at lower risk of LR based on MTV_pre_, higher MTV_mid_ was associated with LR (*P* = 0.001).

**Conclusion:**

Per-lesion, larger MTV_pre_ and MTV_mid_ predicted for increased risk of LR. MTV_mid_ was more highly predictive of LR than MTV_pre_ and if validated may allow for further discrimination of high-risk lesions at mid-RT informing dose painting strategies.

## Introduction

Lung cancer remains the second most commonly diagnosed malignancy among both men and women and is the number one cause of cancer related death [1]. Approximately a third of patients with non-small cell lung cancer (NSCLC) are diagnosed with stage III disease [2, 3], which is commonly treated with definitive chemoradiation and has a median overall survival of approximately 24 months [3]. Encouragingly, a recent phase III trial has shown the addition of adjuvant durvalumab offers a benefit in overall survival [4]. As systemic therapies improve and potentially eradicate distant micrometastases, highly effective local treatments may become increasingly crucial.

Definitive chemoradiation is the standard of care treatment for unresectable stage III NSCLC, but a large proportion of patients will experience local recurrence after definitive local treatment. The standard dose arm from the RTOG 0617 trial (60 Gy) showed a local recurrence rate of 16.3 and 30.7% at one and 2 years, respectively, and dose escalation to 74 Gy did not improve locoregional control [3].

Several groups have investigated the utility of ^18^F-FDG PET-CT (PET-CT) in identifying patients and specific lesions at high risk of treatment failure as they may benefit from a more individualized dose strategy with high-risk lesions receiving dose escalation and low-risk lesions receiving dose de-escalation [5–11]. As a method of quantifying the volume of metabolically active cancer, there has been interest in delineating the metabolic tumor volume (MTV, units cc) on PET imaging. Methods to calculate MTV include gradient based methods, which identify the edge of the metabolically active lesion by finding the location of highest signal gradient, and threshold based methods which include all voxels within a volume having SUV higher than a defined threshold. Gradient based methods have been reported as having better correlation with pathologic findings [12]. We previously reported that higher pre-RT MTV (sum of all lesions) was associated with local recurrence, but other pre-RT PET parameters were not, including maximum SUV and total lesion glycolysis (MTV multiplied by the target volume average SUV) [13]. Ohri et al. identified and validated per-lesion, pre-RT MTV as a predictor of local recurrence [11]. In a subsequent small prospective study, they showed that dose-painted RT with conventional radiation (65 Gy) to high-risk lesions and de-escalation (52.5–57 Gy) to low-risk lesions yields low rates of local recurrence in high-risk (9%) and low-risk (3%) lesions [14].

Beyond pre-RT imaging, PET-CT scans collected in the middle of radiation treatment (mid-RT PET-CT) may assess initial treatment response and provide additional discrimination of high-risk lesions at a time when the treatment plan can be adapted [9, 13, 15]. In a recent phase II trial, target areas with residual metabolic activity on mid-RT PET-CT were selectively boosted, with the patients having a promising local recurrence rate of 18% at 2 years [15]. While a prior study from our group showed that mid-RT MTV of the entire target volume, including primary tumor and involved lymph nodes, is correlated with local recurrence, to our knowledge, no group has studied whether *per-lesion* mid-RT MTV is associated with local recurrence [13].

At our institution we routinely obtain both pre- and mid-RT PET-CT for stage III NSCLC patients. Using institutional data, we sought to externally validate the findings of Ohri et al. regarding pre-RT per-lesion PET appearance, and extend them to mid-RT PET [11]. Therefore, we investigated per-lesion, pre- and mid-RT MTV as biomarkers of local recurrence for these patients, and to our knowledge, report the largest study of this kind.

## Material and methods

### Patients

With institutional review board (IRB) approval, we conducted a retrospective review of our institutional database for all patients with stage III (AJCC 7th edition) non-small cell lung cancer who received definitive radiation (RT, dose≥60 Gy) from June 2006–March 2018 (*n* = 210) and had pre- and mid-RT PET-CT scans. Mid-RT PET-CTs were performed around halfway through RT. Patients who received surgical resection or induction chemotherapy were excluded. Two patients who received adjuvant durvalumab were excluded. Patients enrolled on the RTOG 1106 trial were excluded (NCT01507428). This left 111 patients who met our inclusion criteria.

### Treatment and follow up

We have previously described our institutional treatment protocol [13]. Patients received staging in concordance with the National Cancer Center Network guidelines, usually with confirmation of nodal involvement by endobronchial ultrasound-guided fine needle aspiration. Only ultrasound-suspicious nodes were biopsied, as opposed to comprehensive sampling of all nodal stations. For radiation planning, patients underwent PET-CT simulation with 4D-CT. After confirming patients had serum glucose < 180 mg/dL, they received 12–18 mCi ^18^F-FDG 45–60 min prior to their scan. Before 2013, PET-CT scans were performed on a GE scanner (Milwaukee, WI) with PET data reconstructed with an ordered set expectation maximization algorithm. Subsequently, PET-CT scans were performed on a Siemens Biograph mCT scanner (Siemens, Erlangen, Germany) with PET data reconstructed with time-of-flight using point-spread function modeling. Primary and nodal gross tumor volumes (GTV) were delineated with expansion to internal target volumes (ITV) based on 4D-CT motion. There was no explicit GTV to clinical target volume (CTV) expansion to cover microscopic spread, as most patients were treated with modest dose intensification and the natural dose fall-off provided some coverage for microscopic spread. Elective nodal irradiation was not performed. A final expansion margin was added to the ITV to generate the PTV, generally 5 mm. Plans were normalized to obtain 95% coverage of the PTV with 100% of the prescription dose. Patients in this cohort who received radiation concurrent with systemic therapy most often received weekly carboplatin and paclitaxel. Radiation was delivered via intensity modulated radiation therapy (IMRT) with 6 MV photons using multiple static fields (typically 6–7 fields) or volume modulated arc therapy (VMAT). Daily kV orthogonal films were used for alignment. Patients underwent mid-RT PET-CT imaging halfway through treatment.

Patients were seen in follow up visits at 3-month intervals during the first 2 years after RT with CT or PET-CT imaging performed at each visit and as indicated thereafter. Per institutional standard practice, three-month post-treatment scan was usually a PET/CT, and subsequent imaging was with contrast-enhanced chest CT, with PET-CT ordered only for abnormal findings on CT. Esophageal and pulmonary toxicities were scored according to the National Cancer Institute Common Terminology Criteria for Adverse Events (NCI CTCAE) version 4.03.

### PET analysis

Each primary tumor or nodal target encompassed within an individually distinct PTV was labeled for comparison between pre-RT and mid-RT PET-CT imaging. PET Edge, a gradient-based method included in MIM software (Cleveland, OH), was used to delineate treated lesions (lung tumors and regional lymph nodes) and measure metabolic tumor volume on pre-RT (MTV_pre_) and mid-RT (MTV_mid_) PET-CTs [12]. ΔMTV was defined as (MTV_mid_-MTV_pre_)/MTV_pre_. The distributions of MTV_pre_ and MTV_mid_ were right-skewed and so were log transformed for analysis. The companion CT was used to help identify cancerous lesions but was not directly used to delineate the MTV.

### Local recurrence

We scored local recurrence (LR) based on biopsy confirmation or according to the following radiographic criteria occurring within the prescription dose planning target volume (PTV):

1. Mass-like increase in size by CT
2. Increased FDG uptake in a focal rather than diffuse pattern.

As patients received surveillance imaging as permitted by provider preference and medical insurance, there was variation in surveillance imaging type and quality. Thus, rather than using a specific absolute or percentage change, we defined significant interval change as that which is beyond the threshold for technical scan variation similar to methods employed by other groups [6]. In scoring local recurrences according to radiographic criteria, when available, we did not apply these criteria to isolated imaging scans but evaluated findings in the context of progression on serial imaging or in combination with recurrence in other sites. Out-of-field recurrence was defined as tumor regrowth outside of the prescription dose radiation volume (i.e., any recurrence that was not a LR by our definition).

### Statistical analysis

Median follow up was calculated using the reverse Kaplan-Meier method. Receiver operating characteristic (ROC) curves, adjusted for the competing risk of death were constructed using the R package ‘timeROC’ [16]. The R package ‘maxstat’ was used to determine optimal thresholds for the outcome LR with bootstrap resampling [17]. The cumulative incidence of LR was adjusted for the competing risk of death. When measuring the cumulative incidence of LR per patient, any LR was considered an event. We adjusted for baseline characteristics in multivariable analyses (MVA) using forward selection for patient factors meeting significance of *P*< 0.05 on univariable analysis. As the data had a clustered structure with multiple target lesions per patient, MVA was performed using competing risk regression for clustered data using the R package ‘crrSC’ [18]. Statistical analysis was performed using R version 3.6 (Vienna, Austria). All *p*-values were two-sided and considered significant at *P* < 0.05.

## Results

We identified 111 patients with 387 lesions (112 lung tumors and 275 lymph nodes). Median follow-up was 38.7 months (range, 0.1–109.2 months). As demonstrated in Table 1, patients had median age 68 years, 69.4% were male, 46.8% had adenocarcinoma, and 39.6% had squamous cell carcinoma. 95.5% received concurrent chemotherapy with 78.4% (*n* = 87) receiving concurrent carboplatin/paclitaxel (Supplemental Data). Median radiation dose was 66 Gy (range, 60–74 Gy) and median dose per fraction was 2 Gy (range, 1.8–3 Gy). The most common schedule was 66 Gy in 30 fractions (*n* = 36). Mid-RT PET-CT was obtained at a median dose of 34 Gy (interquartile range [IQR], 30–36 Gy) or at a median proportion of total dose of 50% (IQR, 46–55%). Primary tumor lesions were significantly larger than lymph node lesions when measured on pretreatment PET (median MTV_pre_ of 25.2 vs 1.5 cc, respectively, *P* < 0.0001) and mid-treatment PET (median MTV_mid_ of 14.5 and 0.8 cc, respectively, *P* < 0.0001).

**Table 1:** Baseline patient characteristics

Parameter	Cohort (***n*** = 111)
Median age	68 (42–90) years
Male	77 (69.4%)
ECOG PS	
0	13 (11.7%)
1	70 (63.1%)
2	26 (23.4%)
3	2 (1.8%)
Stage	
IIIA	60 (54.1%)
IIIB	51 (45.9%)
Histology	
Adenocarcinoma	52 (46.8%)
Squamous Cell Carcinoma	44 (39.6%)
Large Cell	3 (2.7%)
Adenosquamous	2 (1.8%)
NOS	10 (9.0%)
Median RT dose	66 (60–74) Gy
60–66 Gy	84 (75.7%)
> 66–74 Gy	27 (24.3%)
Concurrent chemotherapy	106 (95.5%)
Median follow up, months	38.7 (0.1–109.2)

*Abbreviations*: *ECOG PS* performance score, *NOS* not otherwise specified

* Continuous variables are shown with range and categorical variables with percentages in parenthesis

**Fig. 1:**
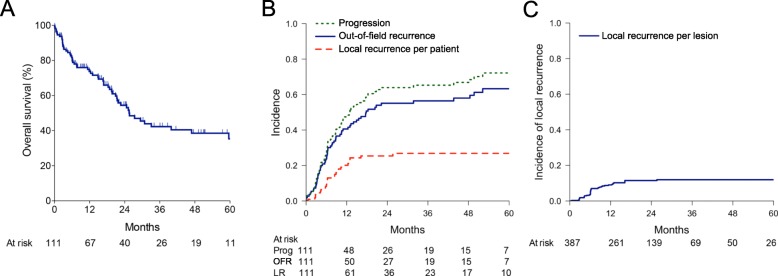
**a**. Overall survival for 111 patients with locally advanced NSCLC. **b**. Cumulative incidence of any progression (green-dased curve), out-of-field recurrence (blue curve), and local recurrence per patient (red-dashed curve). **c**. Cumulative incidence of per-lesion local recurrence for primary tumor and nodal target volumes receiving definitive radiation

**Fig. 2:**
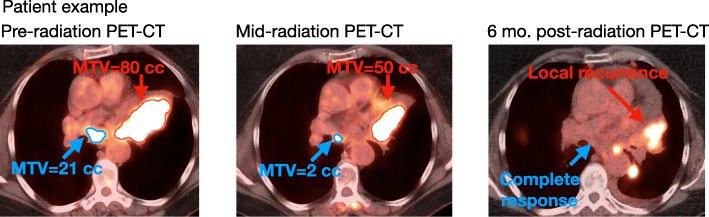
Representative patient example demonstrates per-lesion method of measuring MTV on fused PET-CT images. As shown, a nodal lesion had a complete response to chemoradiation while the primary tumor had large residual MTV at mid-radiation PET-CT with subsequent local recurrence

Overall survival (OS) was 72.7% (95% CI = 63.1–80.2%) at 1 year, 54.5% (95% CI = 43.7–64.1%) at 2 years, and 42.3% (95% CI = 31.3–52.9%) at 3 years (Fig. 1a). Median survival was 25.6 months. Cumulative incidence of non-cancer death was 11.2% (95%CI = 6.5–15.9%) at 3 years (Supplemental Figure 1). Cumulative incidence of LR per patient adjusted for the competing risk of death was 26.8% at 3 years (Fig. 1b**,** 95%CI = 18.4–35.2%). Excluding the five patients who did not receive concurrent chemotherapy, there was a very similar 3-year LR rate of 27.2% (95% = 18.2–36.1%). When scoring LR, 20 of 28 patients (71.4%) had pathologic evidence of recurrence with the remainder being scored by radiographic criteria alone (see Materials and Methods). Cumulative incidence of LR per target lesion adjusted for the competing risk of death for the entire cohort was 11.9% at 3 years (Fig. 1c**,** 95%CI = 10.2–13.6%) and was 11.4% (95% = 9.6–13.2%) for those that received concurrent chemotherapy. Figure 2 shows a representative patient demonstrating our method of measuring MTV on a per lesion basis. This patient experienced a complete response at a nodal target with a small MTV_mid_ and a LR at the primary tumor lesion with a large MTV_mid_. For patients who experienced LR, 10 of 28 (35.7%) experienced isolated LR without distant metastasis. No patient developed LR prior to observed distant recurrence, and 10 patients developed LR concurrent with discovery of distant metastases (15.6% of patients with distant recurrence).

**Fig. 3:**
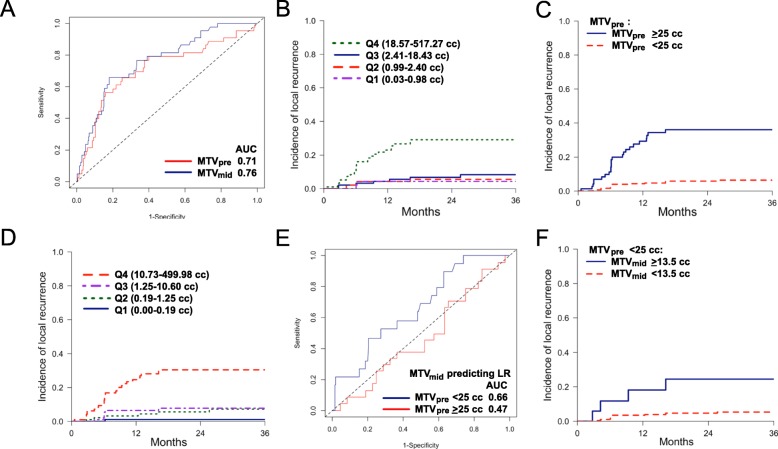
**a**. Competing risk ROC for MTV_pre_ (AUC = 0.71) and MTV_mid_ (AUC = 0.76) predicting local recurrence, adjusted for competing risk of death at 3-years post-treatment. **b**. Cumulative incidence of local recurrence by MTV_pre_ size quartiles. **c**. Cumulative incidence of local recurrence stratified by MTV_pre_ ≥ 25 and < 25 cc . **d**. Cumulative incidence of local recurrence by MTV_mid_ size quartiles. **e**. ROC for MTV_mid_ predicting local recurrence, adjusted for competing risk of death for the subset with MTV_pre_ ≥ 25 cc (AUC = 0.47) and the subset with MTV_pre_ < 25 cc (AUC = 0.66) 36 months post-treatment. **f**. Cumulative incidence of local recurrence for all lesions with MTV_pre_ < 25 cc showing those with MTV_mid_ ≥ 13.5 cc versus < 13.5 cc have 24-month LR rates of 24.5% versus 5.3%, respectively

We sought to determine whether MTV_pre_, MTV_mid_ or both were associated with LR. By ROC, MTV_mid_ (AUC = 0.76) had a slightly higher AUC value than MTV_pre_ (AUC = 0.71) at 3 years post-treatment adjusted for the competing risk of death (Fig. 3a). We next performed univariable competing risk regression adjusted for the competing risk of death and within-patient clustered data, and found MTV_pre_, MTV_mid_, and lesion location (lung primary versus lymph node) were significantly associated with local recurrence while histology and ΔMTV were not (Table 2). Because MTV_pre_ and MTV_mid_ are collinear variables, we performed two MVAs selecting either MTV_pre_ or MTV_mid_ as the MTV variable. When adjusting for lesion location in MVA, we observed that MTV_pre_ (HR = 2.01, 95% CI = 1.25–3.23, *P* = 0.004) and MTV_mid_ (HR = 2.12, 95% CI = 1.47–3.08, *P* = 7.1e-5) were significantly associated with LR (Table 2). Lesion location was not associated with LR in either multivariable model.

**Table 2:** Univariable and multivariable competing risk regression accounting for clustered analysis and competing risk of death

		Local recurrence (*n* = 45 events)		
Variable		Univariable	MVA1	MVA2
Age	HR	1.00		
	95% CI	0.96–1.05		
	*P*	0.84		
ΔMTV	HR	1.09		
	95% CI	0.96–1.24		
	*P*	0.19		
Log(MTV_pre_)	HR	**2.43**		**2.01**
	95% CI	**1.56–3.78**		**1.25–3.23**
	*P*	**8.4e-5**		**0.004**
Log(MTV_mid_)	HR	**2.33**	**2.12**	
	95% CI	**1.61–3.37**	**1.47–3.08**	
	*P*	**6.8e-5**	**7.1e-5**	
Primary Lesion (non-nodal target)	HR	**3.60**	1.37	1.79
	95% CI	**1.86–6.95**	0.67–2.79	0.86–3.72
	*P*	**0.0001**	0.38	0.12
RT dose	HR	1.06		
	95% CI	0.97–1.16		
	*P*	0.17		
Histology				
Adenocarcinoma (ref)	HR	–		
	95% CI	–		
	*P*	–		
SCC	HR	1.89		
	95% CI	0.80–4.46		
	*P*	0.15		
All others	HR	1.27		
	95% CI	0.30–5.45		
	*P*	0.75		

Abbreviations: *MVA* multivariable analysis; *MTV*_*pre*_ pretreatment metabolic tumor volume; *MTV*_*mid*_ mid-treatment metabolic tumor volume; *RT* radiation therapy; *SCC* squamous cell carcinoma; *ref*. reference

As demonstrated in Fig. 3b, lesions with larger MTV_pre_ had a higher risk of LR. When performing threshold analysis for MTV_pre_ using the log-rank statistic for the outcome LR, we obtained an optimal value of ≥25.4 cc (95%CI = 5.77–42.52 cc, 1000 bootstrap resampling). We obtained the same threshold when excluding the 5 cases that received RT alone. Intriguingly, this value was nearly identical to that reported by Ohri et al., and we selected the validated 25 cc threshold for further analysis (there were no lesions with MTV_pre_ between 25.0 and 25.4 cc) [11]. We observed the 3-year cumulative incidence of LR per lesion was 6.4% versus 36.0% for lesions with MTV_pre_ < 25 cc versus ≥25 cc, respectively (Fig. 3c**,***P* = 7.5e-11).

Investigating the relationship between MTV_mid_ and LR similarly demonstrated that lesions with larger MTV_mid_ had a higher risk of LR (Fig. 3d). When performing threshold analysis for MTV_mid_ using the log-rank statistic for the outcome LR, we obtained an optimal value of ≥13.5 cc (95%CI = 0.00–24.85 cc, 1000 bootstrap resampling).

As an exploratory analysis, we assessed the predictive value of MTV_mid_ among the high- and low-risk subsets defined by Ohri et al. and from our results, i.e. lesions with MTV_pre_ ≥ 25 cc and MTV_pre_ < 25 cc, respectively. On univariable analysis, MTV_mid_ remained predictive of LR for the subgroup of lesions with MTV_pre_ < 25 cc (AUC = 0.66) but not for lesions with MTV_pre_ ≥ 25 cc (AUC = 0.47; Fig. 3e). Among lesions with MTV_pre_ < 25 cc, cumulative incidences of local recurrence for lesions with MTV_mid_ < 13.5 cc and MTV_mid_ ≥ 13.5 cc were 5.3 and 24.5% at 3 years, respectively (Fig. 3f). In the subset of lesions with MTV_pre_ < 25 cc, increasing MTV_mid_ was significantly associated with higher risk of LR (HR = 1.92, 95% CI = 1.28–2.28, *P* = 0.002). In the subset of lesions with MTV_pre_ ≥ 25 cc, MTV_mid_ was no longer significantly associated with higher risk of LR (Supplemental Tables 1 and 2).

### Toxicity

Fifty-three (47.7%) patients experienced grade ≥ 2 pulmonary or esophageal toxicity including 44 cases of grade 2 esophagitis, 16 cases of grade 2 radiation pneumonitis, 1 case of grade 3 esophagitis, 3 cases of grade 3 radiation pneumonitis, and 1 case of grade 5 radiation pneumonitis

## Discussion

In this study, we report per-lesion outcomes for patients with stage III NSCLC following definitive chemoradiation, and we show that MTV_pre_ and MTV_mid_ predict per-lesion LR. These findings externally validate the results of Ohri et al., and extend them to mid-treatment imaging [11]. We focused on MTV rather than other PET parameters because we previously observed that only pre-RT MTV had a significant association with LR as compared to other PET parameters including maximum SUV and total lesion glycolysis [13]. Additionally, we did not previously find association between LR and mid-RT maximum SUV or the ratio of mid-RT maximum to the pre-RT value.

Previous efforts to intensify locoregional treatment have been unsuccessful, as seen with RTOG 0617, which demonstrated increased toxicity without a local control benefit when uniform RT dose escalation was applied to all target volumes [3]. However, recent retrospective and prospective studies have demonstrated the feasibility of adapting dose to individual lesions, which may allow for dose escalation and de-escalation of lesions at high and low risk of LR, respectively [11, 15]. Reliable prognostic factors of which lesions are most likely to recur and therefore benefit from treatment intensification remain to be determined but PET-CT based metrics are promising. Prospective studies, including RTOG 1106 (NCT01507428), are evaluating dose escalation of FDG-avid lesions on mid-RT PET-CT [15]. Other prospective trials tailor either dose escalation of the primary tumor or region inside the primary tumor with > 50% SUV_max_ on pre-RT PET-CT (NCT01024829), or de-escalation of lesions with MTV_pre_ < 25 cc [14, 19, 20]. We have previously demonstrated the prognostic ability of total tumor mid-RT PET-CT parameters in predicting locoregional or distant progression, and others have shown an association between mid-RT PET-CT and overall survival [13, 21]. Our current findings lend support to using mid-RT PET-CT for adaptive RT strategies to primary lung tumors and/or regional lymph nodes on a per-lesion level.

We observed a per-patient cumulative incidence of LR of 26.8% at 3 years when accounting for the competing risk of death, an important consideration due to the poor prognosis of patients with stage III lung cancer. Our median overall survival of 25.6 months is comparable to the rate reported by others, suggesting our cohort has a comparable observation period for LR with similar competing risk of death [3, 15, 22]. Overall, our LR rates are similar to or better than those reported in the literature with estimates ranging from 18 to 31% [3, 6, 11, 22]. This improvement is notable in light of the smaller tumor margins that we use without explicit expansion for microscopic extension (CTV = GTV), and may be explained by our routine use of 4D CT and PET-CT simulation, daily image-guidance, respiratory motion management as indicated, and modestly escalated prescription doses.

When analyzed per lesion, we find that both larger MTV_pre_ and MTV_mid_ are predictive of increased risk of LR. We externally validated findings by Ohri et al. in showing a threshold of MTV_pre_ ≥ 25 cc has high specificity in predicting recurrence (albeit with a wide confidence interval), and also add that MTV_pre_ as a continuous variable is predictive of LR. In the cohort reported by Ohri et al., a large subset of lesions with MTV_pre_ < 25 cc (34% of the total target lesions) received < 60 Gy and there was no association between LR and RT dose [11]. This prompted their prospective investigation of dose de-escalation for tumors with low MTV_pre_. When evaluated in a small prospective study, this group found low rates of local recurrence (3%) in low-risk lesions (MTV_pre_ < 25 cc) treated with a reduced dose of 52.5–57 Gy, but high-risk lesions treated with conventional RT had a higher risk of recurrence (9%) [14].

Others have also reported the prognostic utility of pre-treatment tumor size in predicting LR including a secondary analysis of RTOG 0235, which demonstrated increasing total MTV_pre_ (composite of all lesions) was significantly associated with LR [8]. Additionally, a large cohort from the Netherlands found that tumor volume was the only significant predictor of local recurrence [22]. This led to the development of the PET-boost trial (NCT01024829) which evaluated dose-escalation to entire primary tumors or to regions of high pre-RT SUV_max_ within the primary tumor but not involved lymph nodes [19, 20]. However, given that lesion location was not predictive of LR in our study, we hypothesize that MTV_pre_ and MTV_mid_, which are generally smaller for lymph nodes, are more reflective of the risk of LR and therefore better biomarkers for dose-adapted RT.

We identified that MTV_mid_ may have greater predictive utility compared with MTV_pre_ and may provide additional discrimination of lesions at risk for LR when considered together with MTV_pre_. While MTV_mid_ correlates with initial tumor size and MTV_pre_, we hypothesize that MTV_mid_ contributes predictive information not captured in MTV_pre_. MTV_mid_ may reflect the radioresistant volume of tumors for which current definitive RT doses are insufficient and inherent patient and tumor biologic factors demonstrating worse response to treatment. A prospective trial demonstrated an encouraging 2-year in-field local control rate of 82% with mid-RT PET-CT informed dose escalation to residual tumor with doses up to 86 Gy. In this study, the rate of grade 3 radiation pneumonitis was tolerable at 7% [15]. We await results from the randomized RTOG 1106 in evaluating the benefit of selective target volume dose escalation (NCT01507428).

The question remains as to whether MTV_pre_ and MTV_mid_ can predict LR better together versus individually. We found that while MTV_pre_ and MTV_mid_ were each highly predictive of LR, ΔMTV (MTV_mid_-MTV_pre_)/MTV_pre_ was not associated with LR. There may be a more complicated relationship between MTV_mid_ and MTV_pre_ in predicting tumor response, which may depend on tumor histology, RT dose, and/or timing of mid-RT PET-CT. Interestingly, a study of patients with stage I-III NSCLC found that a greater reduction in mid-RT MTV was associated with worse survival in patients treated with conventional RT, but improved survival in those treated with PET-adapted dose escalation to the mid-RT residual tumor volume. While counterintuitive, this lends further support to the study of mid-RT PET-adapted RT in RTOG 1106 [21].

To explore how to use both MTV_pre_ and MTV_mid_ as biomarkers of LR, we investigated the predictive utility of MTV_mid_ in the high-risk (MTV_pre_ ≥ 25 cc) and low-risk (MTV_pre_ < 25 cc). We found that MTV_mid_ was predictive of LR in the subset of low-risk lesions with MTV_pre_ < 25 cc. This suggests lesions with MTV_pre_ < 25 cc that either resolve or have significantly reduced MTV_mid_ would be amenable for lower target dose as explored by others [14]. In contrast, low-risk lesions with MTV_pre_ < 25 cc and MTV_mid_ ≥ 13.5 cc have a higher risk of local recurrence and may not benefit from de-escalated RT. Given the acceptable toxicity of dose escalation to sites of residual FDG-avidity at mid-RT PET-CT, these lesions, as well as those with MTV_pre_ ≥ 25 cc, may actually benefit from dose escalation [15]. In future trials, MTV_mid_ may be able to identify small, seemingly low-risk lesions that are at increased risk of local recurrence based on their underlying biology and radiosensitivity. For example, in Ohri et al’s prospective study of dose painting RT, 2 of the 3 low-risk lesions that recurred were presumed low risk with very small MTV_pre_ of 2 cc [14].

We did not find that MTV_mid_ provided additional prognostic utility for lesions already identified as high risk for LR with MTV_pre_ ≥ 25 cc. Given that the threshold of MTV_pre_ ≥ 25 cc was identified based on the predictive capacity of MTV_pre_ alone, it is possible that we could determine better thresholds leveraging information from MTV_pre_ and MTV_mid_. However, the low toxicity of higher RT doses to individual sites defined as high-risk by MTV_pre_ and by FDG-avidity at mid-RT PET-CT suggest that a higher RT dose to lesions with MTV_pre_ ≥ 25 cc may be associated with an acceptable toxicity profile [11, 15].

Limitations of our analysis include its retrospective nature and fairly short follow-up time. Our results should be applied with caution to populations that represented a minority of our dataset, such as patients who did not receive chemotherapy. Many patients in our series were treated to slightly higher than standard dose per fraction (2.2Gy), which could affect local recurrence rates. There may be difficulties for other radiation oncology departments to obtain mid-RT PET-CT scans. Finally, our specific MTV thresholds and rates of LR in our cohort may not be representative for patients receiving adjuvant durvalumab, a treatment that became standard of care after the patients in our cohort were treated. The patterns of failure for patients enrolled in the PACIFIC trial were presented in abstract form in 2019 but did not include detailed information regarding the rate of ‘in-field’ recurrences after RT [23]. Nevertheless, there was an 11.5% absolute reduction in thoracic recurrences as first recurrences for patients receiving adjuvant durvalumab compared with no further treatment, suggesting local control may be improved with adjuvant immunotherapy. More detailed patterns of failure studies assessing recurrence location relative to radiotherapy volumes for patients receiving definitive chemoradiotherapy with adjuvant durvalumab are required.

In summary, our results show larger MTV_pre_ and MTV_mid_ are associated with increased risk of LR, and MTV_mid_ may hold higher predictive utility, particularly in the setting of small lesions. If validated in larger cohorts, this may be the basis for designing adaptive dose painting strategies to maximize therapeutic index. Specifically, our data support prospective study of dose escalation to high-risk lesions with MTV_pre_ ≥ 25 cc. However de-escalation of lesions with MTV_pre_ < 25 cc should be performed cautiously, as lesions with more sluggish response (MTV_mid_ ≥ 13.5 cc) may have a higher risk of local recurrence.

## Supplementary information

**Additional file 1: Table S1.** Subset of lesions with MTVpre< 25 cc. Competing risk regression accounting for clustered analysis and competing risk of death. **Table S2.** Subset of lesions with MTVpre ≥25 cc. Competing risk regression accounting for clustered analysis and competing risk of death.

**Additional file 2: Figure S1.** Incidence of non-cancer death.

**Additional file 3. Supplemental Figure 1.** Incidence of non-cancer death.

## Data Availability

Data is protected health information and will not be provided.
