# Supplementary material for: Predicting per-lesion local recurrence in locally advanced non-small cell lung cancer following definitive radiation therapy using pre- and mid-treatment metabolic tumor volume
Source: Radiat Oncol. 2020 May 19;15:114. doi: 10.1186/s13014-020-01546-y (PMC7238662; doi:10.1186/s13014-020-01546-y)
Supplement: Supplementary file 1 — Additional file 1: Table S1. Subset of lesions with MTVpre< 25 cc. Competing risk regression accounting for clustered analysis and competing risk of death. Table S2. Subset of lesions with MTVpre ≥25 cc. Competing risk regression accounting for clustered analysis and competing risk of death. [file 13014_2020_1546_MOESM1_ESM.pdf]

Supplemental Table 1. Subset of lesions with MTV_{pre}<25 cc. Competing risk regression accounting for clustered analysis and competing risk of death.

Variable		Local Recurrence (n=21 events) Univariable
Age	HR<br>95% CI<br>p	1.04<br>0.96-1.13<br>0.35
Log(MTV_{mid})	HR<br>95% CI<br>p	**1.92**<br>**1.28-2.28**<br>**0.002**
Primary Lesion (non-nodal target)	HR<br>95% CI<br>p	2.28<br>0.91-5.66<br>0.08

Abbreviations: MTV_{mid}=mid-treatment metabolic tumor volume.

Supplemental Table 2. Subset of lesions with $MTV_{pre} \geq 25$ cc. Competing risk regression accounting for clustered analysis and competing risk of death.

Variable		Local Recurrence (n=24 events) Univariable
Age	HR<br>95% CI<br>p	0.97<br>0.92-1.01<br>0.11
Log(MTV_{mid})	HR<br>95% CI<br>p	1.23<br>0.47-3.27<br>0.67
Primary Lesion (non-nodal target)	HR<br>95% CI<br>p	1.12<br>0.41-3.09<br>0.83

Abbreviations: MTV_{mid}=mid-treatment metabolic tumor volume.
