# Supplementary figures and images for: Predicting per-lesion local recurrence in locally advanced non-small cell lung cancer following definitive radiation therapy using pre- and mid-treatment metabolic tumor volume
Source: Radiat Oncol. 2020 May 19;15:114. doi: 10.1186/s13014-020-01546-y (PMC7238662; doi:10.1186/s13014-020-01546-y)

**Supplemental Figure 1. Incidence of non-cancer death**

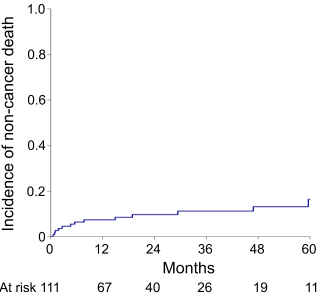

Supplement: Supplementary file 2 — Additional file 2: Figure S1. Incidence of non-cancer death. [file 13014_2020_1546_MOESM2_ESM.pdf]
